# A multivalent biparatopic EGFR-targeting nanobody drug conjugate displays potent anticancer activity in solid tumor models

**DOI:** 10.1038/s41392-021-00666-5

**Published:** 2021-09-03

**Authors:** Jiansheng Fan, Xinlei Zhuang, Xiaoyue Yang, Yingchun Xu, Zhan Zhou, Liqiang Pan, Shuqing Chen

**Affiliations:** Institute of Drug Metabolism and Pharmaceutical Analysis, College of Pharmaceutical Sciences, Zhejiang University, Hangzhou, China

**Keywords:** Drug development, Drug development

**Dear Editor**,

The epidermal growth factor receptor (EGFR), one member of the ErbB family of receptor tyrosine kinases, has been implicated in various epithelial malignancies. As a clinically validated target, both small-molecule tyrosine kinase inhibitors and antibody-based drugs have been approved by regulatory agencies. Anti-EGFR antibody-drug conjugates (ADCs) could kill target tumor cells irrespective of EGFR signaling. However, cancer patients treated with EGFR-targeted antibody therapy eventually develop acquired epitope substitutions such as S492R, G465R, G465E, and I491M, which abolish cetuximab or panitumumab binding and further gives rise to resistance to anti-EGFR ADC therapy.^1^ Our team has constructed a tetravalent biparatopic anti-EGFR nanobody-drug, consisting of two tandemly fused anti-EGFR nanobodies (7D12 and 9G8) targeting two distinct non-overlapping epitopes (9G8-7D12-Fc, abbreviated as 97m). Coupling anti-mitotic agent monomethyl auristatin E (MMAE) to the conservative engineered surface cysteine S7C on 7D12 part of 97m resulting its conjugate, abbreviated as S7 ADC (Fig. 1a and Supplementary Fig. S1a–c). The tetravalent biparatopic ADC demonstrated high conjugation efficiency, small binding interfaces to overcome cetuximab-resistant mutations, enhanced complement-dependent cytotoxicity (CDC), and highly potent antitumor activity. The introduction of E430G in the Fc domain would further boost the complement-mediated immune response of S7 ADC, synergizing with the cytotoxicity of drug payload during cancer treatment.

**Fig. 1:**
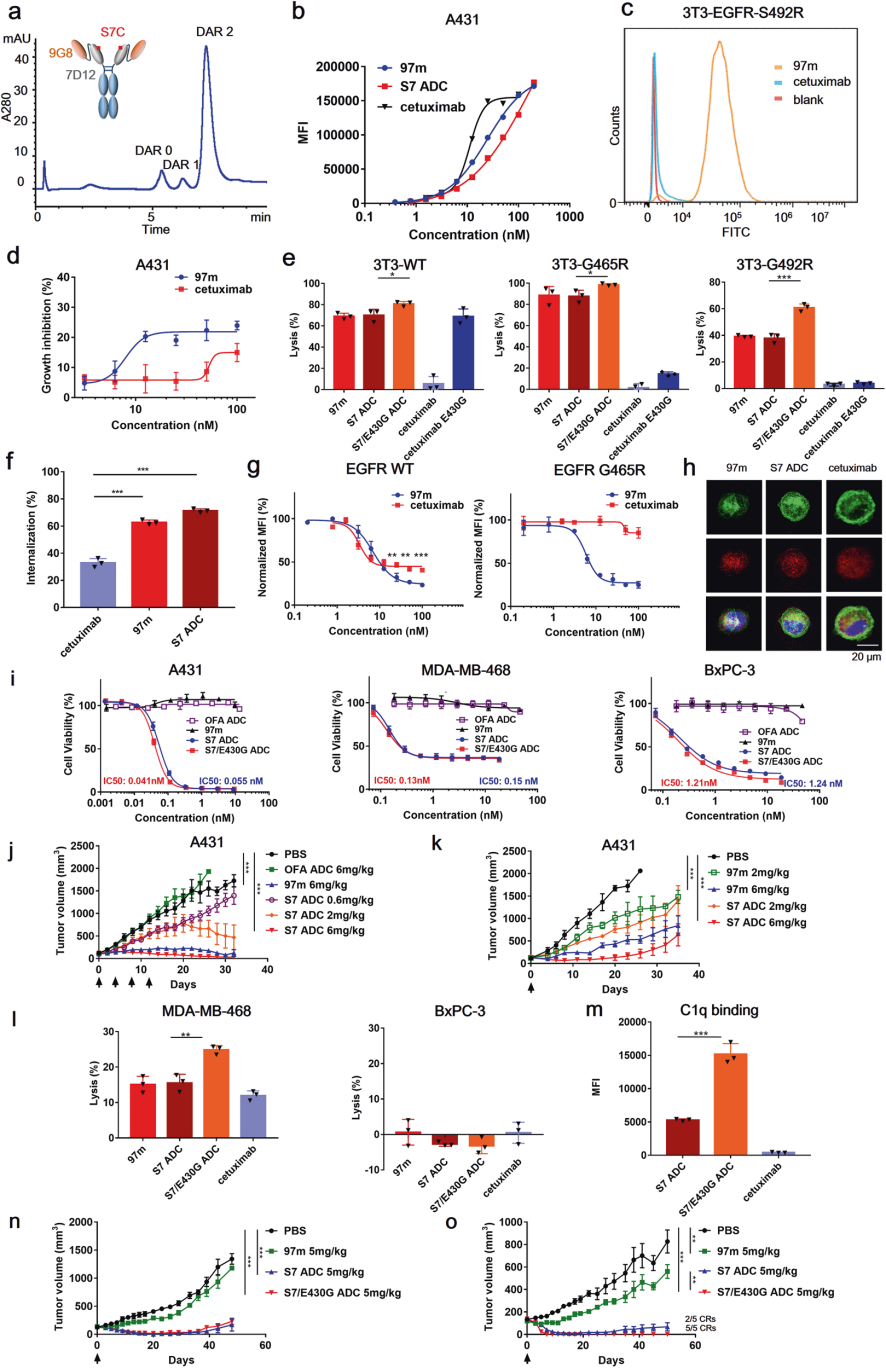
Multivalent anti-EGFR nanobody drug conjugate. **a** Schematic diagram of 97m and Hydrophobic interaction chromatography (HIC) analysis of S7 ADC. **b** Flow cytometry analysis of concentration-dependent binding of cetuximab, 97m and S7 ADC with EGFR-positive A431 cells. **c** Binding ability of biparatopic nanobody 97m and cetuximab with EGFR-S492R NIH-3T3 cells. **d** The inhibition effect of 97m and cetuximab on A431 tumor cell proliferation. **e** CDC activity of biparatopic nanobody and its conjugates on EGFR-wt, EGFR-G465R, and EGFR-S492R NIH-3T3 cells. **f** Internalization of cetuximab, 97m and S7 ADC in A431 cells. **g** Quantification of internalized and degraded EGFR through flow cytometry. **h** Intracellular trafficking and lysosomal localization of cetuximab, 97m and S7 ADC in A431 cells at 37 °C for 1.5 h antibodies (green), lysosomes (red), nucleus (blue). **i** In vitro cytotoxicity of S7 ADC and S7/E430G ADC on A431, MDA-MB-468, and BxPC-3 tumor cells. **j** In vivo antitumor activities of multi-dose 97m and S7 ADC in A431 xenograft models. **k** In vivo antitumor activities of single-dose 97m and S7 ADC in A431 xenograft models. **l** CDC activity of 97m and its conjugates on MDA-MB-468 and BxPC-3 cells. **m** Flow cytometry analysis of C1q deposition on A431 cells in the presence of S7 ADC, S7/E430G ADC, or cetuximab. **n** In vivo antitumor activities of single-dose S7 ADC and S7/E430G ADC in BxPC-3 xenograft models. **o** In vivo antitumor activities of single-dose S7 ADC and S7/E430G ADC in MDA-MB-468 xenograft models

The drug-antibody ratio (DAR) of S7 ADC was calculated by hydrophobic interaction chromatography profile. S7 ADC (DAR = 1.83) exhibited a homogeneous conjugation profile, suggesting the superior accessibility of S7C mutation with respect to site-specific conjugation (Fig. 1a and Supplementary Fig. S2). Both 97m and S7 ADC bound to EGFR-positive A431 cells in a concentration-dependent manner, and showed attenuated binding capacity with A431 cells comparing to cetuximab (Fig. 1b). However, 97m showed binding with various EGFR mutant cell lines which harbored mutations leading to cetuximab resistance, suggesting its potential of delivering toxins to EGFR mutated tumor cells (Fig. 1c and Supplementary Fig. S3).

As a tetravalent form, 7D12 interrupts the EGFR signaling cascade upon binding, while 9G8 stabilizes the tethered conformation of EGFR ECD to sterically prevent it to form dimerization.^2^ 97m was demonstrated to be more effective than cetuximab in inhibiting proliferation of EGF treated A431 cells (Fig. 1d). With two nanobodies recognizing two non-overlapping EGFR epitopes, 97m or S7 ADC alone also elicited potent CDC on NIH-3T3 expressing wildtype EGFR while cetuximab did not show any obvious CDC. We further introduced E430G mutation to the Fc domain of S7 ADC to facilitate hexamer formation through enhancing intermolecular Fc-Fc interactions between cell-bound molecules for the generation of S7/E430G ADC^3^ (Supplementary Fig. S4). S7/E430G ADC also showed higher CDC than S7 ADC on NIH-3T3 expressing wildtype EGFR or harboring cetuximab-resistant mutation (Fig. 1e).

Receptor-mediated endocytosis is an important mechanism implicated in ErbB-targeted immunotherapy. Enhanced internalization and lysosomal trafficking could benefit the uptake of ADC, thus improving its efficacy.^4^ The internalization of 97m and S7 ADC were two times as much as cetuximab (Fig. 1f). In a receptor degradation experiment, 97m significantly reduced the EGFR-eGFP signal of HEK293T cells expressing EGFR-eGFP or EGFR-G465R-eGFP cells (Fig.1g). The intracellular trafficking of antibodies were illustrated by a confocal laser scanning microscope. Comparing to cetuximab, 97m and S7 ADC showed more uptake by A431 within 1.5 h (Fig. 1h). The majority of internalized 97m or S7 ADC were co-localized with lysosomal marker LAMP-2, suggesting they were successfully trafficked to lysosome as expected.

Both rapid internalization and lysosomal degradation of receptor could contribute to the effective ADC delivery and intracellular release of payload in tumor cells. S7 ADC treatment induced much higher apoptosis of A431 and MDA-MB-468 cells and triggered a substantial dose-dependent shift of tumor cell cycle from G1 phase to G2/M phase with respect to 97m group (Supplementary Fig. S5a, b). A431, MDA-MB-468, BxPC-3 were treated with 97m, S7 ADC, S7/E430G ADC, and ofatumumab-vc-MMAE (anti-CD20 non-binding control ADC, abbreviated as OFA-ADC, Supplementary Fig. S6) for 3 days. S7 ADC and S7/E430G ADC exhibited similar and potent in vitro efficacy on all tested EGFR-positive tumor cell lines, and the introduced E430G mutation did not affect the in vitro efficacy of biparatopic ADC in the absence of complement involvement (Fig. 1i).

In vivo antitumor efficacy of S7 ADC was tested on A431 xenograft model. Four doses (q4d×4) of S7 ADC or 97m (each at 6 mg/kg) treatment resulted in sustained tumor growth regression in the xenograft mouse model, while non-binding control OFA-ADC showed no effect (Fig. 1j). There was no sign of obvious adverse events in any of the treatment groups (Supplementary Fig. S7). A single dose of S7 ADC or 97m (each at 6 mg/kg) also significantly delayed tumor growth, and S7 ADC showed better in vivo efficacy than 97m (Fig.1k). Therefore, biparatopic nanobodies and its ADC, equipped with two different papratopes and two modes of EGFR inhibition, could exert potent dose-dependent antitumor activity in A431 tumor xenografts. In vivo distribution of Cy5-labeled antibodies or ADC was monitored in A431 and SW480 xenograft models, demonstrating the specific accumulation of antibodies or ADC in both tumor sites (Supplementary Fig. S8). Pharmacokinetic studies were conducted in Balb/c mice, 97 m and its conjugates showed similar half-life and other PK characteristics in comparison with cetuximab (Supplementary Fig. S9 and Supplementary Table S1).

Since cetuximab has benefited from CDC on in vivo efficacy,^5^ we hypothesize that enhanced CDC would further boost the complement-mediated immune response of ADC, synergizing with the cytotoxicity of drug payload during cancer treatment. We also evaluated the CDC activity of antibody on another two EGFR-positive tumor cell lines, MDA-MB-468 and BxPC-3. BxPC-3 was used as a negative control cell. Since it is a complement-insensitive cell line with high expression of CD59, which is a membrane protein that could protect cells from CDC. S7/E430G ADC exhibited significantly stronger CDC than S7 ADC on MDA-MB-468 cells, while none of the antibodies or their conjugates showed detectable CDC on BxPC-3 cells as expected (Fig. 1l). On the other hand, C1q binding assay was used to directly evaluate the potential of CDC enhanced ADC on initiating the C1-complex formation. Both ADCs showed superior C1q binding avidity than cetuximab (Fig. 1m). To examine whether the enhanced CDC could potentiate the antitumor activity of ADC in vivo, mice bearing BxPC-3 or MDA-MB-468 xenografts were treated with S7 ADC and S7/E430G ADC. In the complement-insensitive BxPC-3 xenograft model, both S7/E430G ADC and S7 ADC efficiently inhibited tumor growth and yet no difference was found between the two ADC groups (Fig. 1n). In another MDA-MB-468 xenograft model, a single injection of S7/E430G ADC quickly shrunk the tumor volume within 5 days and achieved 5 complete remissions (CR) until the end of the experiment (Fig. 1o). One single dose of S7 ADC eliminated the tumor of all 5 mice in 15 days, but tumor relapse occurred in 3 out of 5 mice at the end of the experiment. Taken together, the above results demonstrated that CDC could further enhance the therapeutic efficacy of ADC, suggesting the presence of a synergistic effect.

In conclusion, combinations of mechanistically different nanobodies that recognize non-overlapping epitopes could potentiate the efficacy of anti-EGFR antibody therapy. Besides, these biparatopic antigen-targeting modules could evade targeting resistance hotspot mutations (e.g., S492, G465, K467, K489, and I492) to cetuximab or panitumumab. Our findings indicate the advantage of nanobodies for constructing multivalent antibodies drug conjugate, and highlight the CDC enhanced biparatopic nanobody drug conjugate as a novel ADC with promising therapeutic efficacy for treatment of EGFR-overexpressing solid tumors with or without acquired point mutations.

## Supplementary information

Supplementary information

## Data Availability

The data sets used for the current study are available from the corresponding author upon reasonable request.
