# Supplementary material for: A multivalent biparatopic EGFR-targeting nanobody drug conjugate displays potent anticancer activity in solid tumor models
Source: Signal Transduct Target Ther. 2021 Sep 3;6:320. doi: 10.1038/s41392-021-00666-5 (PMC8413295; doi:10.1038/s41392-021-00666-5)
Supplement: Supplementary file 1 — Supplementary information [file 41392_2021_666_MOESM1_ESM.docx]

**Supplementary Materials**

# A multivalent biparatopic EGFR-targeting nanobody drug conjugate displays potent anticancer activity in solid tumor models

# Jiansheng Fan, Xinlei Zhuang, Xiaoyue Yang, Yingchun Xu, Zhan Zhou, Liqiang Pan** and Shuqing Chen**

# Institute of Drug Metabolism and Pharmaceutical Analysis, College of Pharmaceutical Sciences, Zhejiang University, Hangzhou 310058, China

# *Corresponding authors: panliqiang@zju.edu.cn; [chenshuqing@zju.edu.cn](mailto:chenshuqing@zju.edu.cn)

**This file includes:**

Materials and Methods

Supplementary Figure S1 –S9

Supplemental Tables S1

**Materials and methods**

**Cell lines and reagents**

The epidermoid carcinoma cell line A431, pancreatic carcinoma cell line BxPC-3, breast carcinoma cell line MDA-MB-468 and NIH 3T3 were purchased from Stem Cell Bank, Chinese Academy of Sciences (Shanghai, China). A431, MDA-MB-468，NIH 3T3 and SW48 cells were maintained in DMEM. BxPC-3 cells were cultured in RPMI-1640. All the medium was supplemented with 10% fetal bovine serum (FBS, Gibco), 100 U/mL penicillin and 100 μg/mL streptomycin in a humidified incubator with 5% CO_2_ at 37°C.

NIH 3T3 cells were transfected with wildtype or mutant EGFR plasmids by Lipofectamine 3000 (Invitrogen). NIH 3T3 cell lines stably expressing EGFR wildtype, S492R or G465R mutant were established by limited dilution approach plus puromycin selection.

HEK293T cells were transduced with vectors containing C-terminal enhanced GFP (eGFP) fusion of full-length EGFR or EGFR-G465R. Stably cells were selected with puromycin.

MC-val-cit-PAB-MMAE (vc-MMAE) was obtained from Levena Biopharm (Nanjing, China).

Normal human serum (NHS) collected under informed consent from healthy donors was used as the source of complement. Heated inactivated NHS was obtained by incubation of the serum at 56 °C for 30 min.

**Preparation of anti-EGFR antibodies and biparatopic nanobodies**

Genes encoding heavy and light chains of anti-CD20 mAb ofatumumab or anti-EGFR mAb cetuximab were subcloned into GC-rich eukaryotic expression vector pMH3 (AmProtein, Hangzhou, China) to obtain pMH3-OFA-H, pMH3-OFA-L, pMH3-CTX-H and pMH3-CTX-L plasmids, respectively. The pMH3-OFA-H and pMH3-OFA-L plasmids were transiently co-transfected into human embryonic kidney cells (HEK 293) to express ofatumumab. Cetuximab was expressed via transfection of pMH3-CTX-H and pMH3-CTX-L plasmids into HEK293 cells. To generate biparatopic nanobodies, Nanobody 9G8 (PDB ID: 4KRP) was genetically fused to N-terminus of nanobody 7D12 (PDB ID: 4KRL) via a (G4S)_3_ linker. The 9G8-7D12 tandem nanobody-encoding gene was then subcloned into upstream of human IgG1 Fc (hinge, CH2 and CH3 domains) in pMH3-IgG1 expression vector for the expression of final biparatopic nanobodies. The S7C and E430G mutation were introduced into nanobody 7D12 and IgG Fc region of biparatopic nanobodies by PCR-mediated mutagenesis, respectively. Biparatopic nanobodies (9G8-7D12-Fc, abbreviated as 97m) and mutants (9G8-7D12 (S7C)-Fc and 9G8-7D12 (S7C)-Fc (E430G), abbreviated as 97m-S7C and 97m-S7C/E430G) were expressed in HEK293 cells through transient transfection of their expression plasmids. . Five to seven days post transfection, supernatants were harvested by centrifugation and sterile filtered (0.22 μm). All recombinant proteins were purified by Protein A affinity chromatography (HiTrap Protein A, GE) and buffer exchanged into PBS (pH 7.4) by ultrafiltration. The concentration of purified protein was determined by nanodrop at the absorbance of 280 nm.

**Evaluation of EGFR expression level by flow cytometry**

EGFR expression level on various cell lines was analyzed by flow cytometry. Briefly, around 5×10^5^ cells were harvested and incubated with biparatopic nanobodies or cetuximab on ice for 30 min. After washing twice with PBS (pH 7.4), cells were immunostained with anti-Human Fc PE-conjugate (Abcam) or goat anti-Human IgG (H+L) FITC-labeled (Beyotime Biotechnology) as secondary antibody for 30 min at 4°C. Excessive antibodies were washed away by ice-cold PBS (pH 7.4). The mean fluorescence intensity (MFI) of cell samples was analyzed on a flow cytometer (ACEA NovoCyte).

**Preparation of ADCs**

The biparatopic nanobodies (97m) cysteine mutant 97m-S7C was adjusted to 3 mg/mL with PBS. Blocking cysteine or glutathione on the introduced cysteine was removed by reduction with 50-fold molar excess Tris (2-carboxyethyl) phosphine (TCEP) and diafiltration. Reduced 97m-S7C was then incubated with 5-fold molar excess dehydroascorbic acid (DHAA) for 2h at 25°C to reoxidize the interchain disulfide bonds, leaving the introduced cysteine reduced. Partially reoxidized 97m-S7C was reacted with 5-fold molar excess vc-MMAE at 4°C overnight. The 97m-S7C-vcMMAE conjugates (abbreviated as S7 ADC) was purified by Protein A affinity column and buffer exchanged to PBS by diafiltration. Other biparatopic ADCs, 97m-S7C/E430G (abbreviated as S7/E430G ADC), were prepared according to the above protocol.

For the control ADC ofatumumab-vc-MMAE (OFA-ADC), ofatumumab was reacted with 5-fold molar excess TCEP for 2h at 37°C. Then the reaction system was incubated with 8-fold vc-MMAE at 4°C for 1h. Finally, OFA-ADC was purified and buffer exchanged to PBS (pH 7.4).

**Hydrophobic interaction chromatography (HIC) Analysis**

Drug-to-antibody ratio (DAR) was determined by HIC-HPLC analysis by using TSk gel Butyl-NPR column (2.5 μm, 4.6 mm×3.5 cm, TOSOH, Japan). Antibody and its conjugates were separated by a 15 min linear gradient elution from buffer A: 1.5 M ammonium sulfate, 25 mM sodium phosphate, pH = 7.0 to buffer B: 75% sodium phosphate 25% isopropanol, pH = 7.0 at 0.8 mL/min.

**Size-exclusion chromatography (SEC) analysis**

Aggregation and purity of antibodies and ADCs were evaluated by size-exclusion chromatography using G3000SWxl size exclusion column (5 μm, 7.8 mm×30 cm, TOSOH, Japan) with mobile phase: 50 mM sodium phosphate, 300 mM sodium chloride (pH=7.0) at 0.6 mL/min.

**Internalization assay and antibody induced EGFR degradation**

A431 cells were incubated with 100 nM antibody or ADC on ice for 30 min. After washing, an aliquot of cells were taken out to start internalization by incubation 37°C for 30 min. After ice-cold or 37°C incubation, Cells were stained with anti-Human Fc PE-conjugate (Abcam), washed and analyzed by flow cytometry. The internalization of antibody or ADC was calculated by formula below:

Internalization% = (1-$\frac{\mathrm{MFI} of sample incubated at 37^{\circ}C}{MFI of control sample incubated at 4^{\circ}C}$)×100%

MFI value was determined by subtracting the untreated control background MFI value.

Antibody induced EGFR degradation was assessed by flow cytometry following 48 h incubation of 293T cells expressing EGFR-eGFP or EGFR-S492R-eGFP with cetuximab and 97m. MFI values were normalized to the eGFP signal of untreated controls.

**Confocal analysis for intracellular localization**

To visualize antibody internalization and lysosomal trafficking, cells were seeded at a density of 1×10^5^ cells/mL and treated with 100 nM antibody and ADC for 1.5 h at 4°C or for 1.5 h and 10 h at 37°C. After washing, cells were fixed by 4% paraformaldehyde for 15 min and then permeabilized with 0.1% Triton X-100, 0.2% bovine serum albumin in PBS for 10 min. Cells were stained with Cy5-labeled goat anti-human IgG (H+L) (Abcam) to visualize the antibody or ADC, or stained by primary rabbit anti-human LAMP-1 (Abcam) and secondary Cy3-labeled goat anti-rabbit IgG (H+L) (Abcam) to visualize the lysosomes after blocking step. The nuclei were stained with DAPI. Fluorescence images were acquired with Olympus FV3000 confocal laser scanning microscope.

**Complement-dependent cytotoxicity assay**

Cell lines were seeded into 96-well plates at a density of 1×10^4^ cells/well in complete culture medium and treated with drugs and 20% NHS at 37°C for 3 – 6 hrs. Cell viability was determined using Cell Counting Kit-8 (CCK-8) from Dojindo according to the manufacturer’s instructions.

**C1q binding assay**

Cells were incubated for 30 min on ice with biparatopic nanobodies or antibodies before addition of 20% human serum. After incubation, cell samples were washed twice with ice cold PBS and stained with FITC-conjugated rabbit anti-C1q (Abcam) to detect bound C1q. All the samples were further incubated at 4°C for 30 min before flow cytometric analysis.

**Inhibition of EGFR-mediated cell proliferation**

Cells were seeded at a density of 5×10^3^ cells/well in 96-well plates. After overnight culture, cells were cultured in DMEM medium supplemented with 1% FBS containing 0.5 nM EGF. After 96 hours incubation with antibodies, cell viability was evaluated by the CCK-8 assay.

**In vitro cytotoxicity of ADCs**

In vitro cytotoxicity assay was performed to evaluate the in vitro efficacy of ADCs. Cells were seeded at a density of 5×10^3^ cells/well in 96-well plates and incubated overnight, followed by treatment with serial concentrations of antibodies or ADCs. After 72 hrs treatment, cell viability was determined by CCK-8 assay following manufacturer’s protocol. The 50% inhibitory concentration (IC50) values were calculated using logistic non-linear regression analysis. Data was analyzed with GraphPad Prism 7 (GraphPad Software Inc., San Diego. CA) and presented as mean ± SD from three independent experiments.

**In vivo anti-tumor efficacy of ADCs**

BALB/c nude mice (female, 6-8 weeks old) were purchased from Slaccas (Shanghai, China), housed under specific pathogen-free conditions in a humidity-controlled environment. A431 xenograft model was generated by subcutaneous injection into the right flank of BALB/c nude mice with 4×10^6^ tumor cells. The treatment was initiated on the fifth day, when the average tumor volume of each group reached 100 mm^3^. Mice were randomly divided into 6 groups (n = 6) and intravenously injected with S7 ADC (0.6, 2, 6 mg/kg), 97m (6 mg/kg), ofatumumab-vc-MMAE (6 mg/kg) and PBS (control group) every 4 days for 4 injections (q4d×4). For single dose study, mice were randomly divided into 5 groups (n = 5) and intravenously injected with S7 ADC (2, 6 mg/kg), 97m (2, 6 mg/kg) and PBS (control group). A tumor volume of 2000 mm^3^ was chosen as endpoint for both experiments after which mice were sacrificed under isoflurane anaesthesia.

Twenty-four hours after the fourth administration of S7 ADC (6 mg/kg), ofatumumab-vc-MMAE (6 mg/kg) or PBS, mice bearing A431 tumors were sacrificed under isoflurane anaesthesia. Organs of mouse xenograft models, i.e., heart, liver, lung and kidney, were harvested and fixed by 10% neutral buffered formalin, paraffin embedded and stained with Hematoxylin and Eosin (H&E) for histopathological examination.

For another solid tumor models, mice were inoculated subcutaneously with BxPC-3 (5×10^6^) or MDA-MB-468 (5.5×10^6^), and were randomized into 4 study groups (n = 5) when the tumor volume reached 100–150 mm^3^. S7 ADC, S7/E430G ADC or 97m was administered as a single intravenous dose of 5 mg/kg. The control group received PBS.

Body weight and tumor volume were monitored two to three times weekly throughout the study period. Tumor volumes were obtained via electronic caliper and calculated according to the following formula: tumor volume (mm^3^) = (length × width^2^) /2.

Data was analyzed by GraphPad Prism 7 (GraphPad Software Inc., San Diego. CA) and presented as mean ± SEM.

**In vivo fluorescence imaging of antibodies and ADC**

Eight-week-old female Balb/c nude mice were used for the in vivo imaging experiment.

The EGFR-positive tumor xenograft mouse model was constructed in Balb/c nude mice by inoculation with two EGFR-positive cell lines A431 (high EGFR expression level) and SW480 (middle EGFR expression level) at the left and right flank, respectively.

When the tumors reached approximately 500 mm^3^, Cy5 labeled cetuximab, 97m and S7 ADC were injected into tumor-bearing mice at a dosage of 5 mg/kg. The in vivo distribution of all samples was observed by Maestro In vivo Imaging System (Cambridge Research Instrumentation Inc., USA). All the images were analyzed by CRi Maestro Image software.

**In vivo pharmacokinetics of antibodies and ADCs**

Eight-week-old female Balb/c mice were divided randomly into four groups and then were administered intravenously with cetuximab, 97m, S7 ADC and S7/E430G ADC (n=4) at a dosage of 5 mg/kg. Blood samples were obtained at different time points (0.5 h, 1 h, 4 h, 8 h, 24 h, 48 h, 96 h, 168 h, and 288 h after injection). The concentrations of antibodies or ADCs were measured by double antibody sandwich ELISA. The pharmacokinetic parameters were determined by DAS 3.0 (BioGuider Co., Shanghai, China) in a non-compartment model.


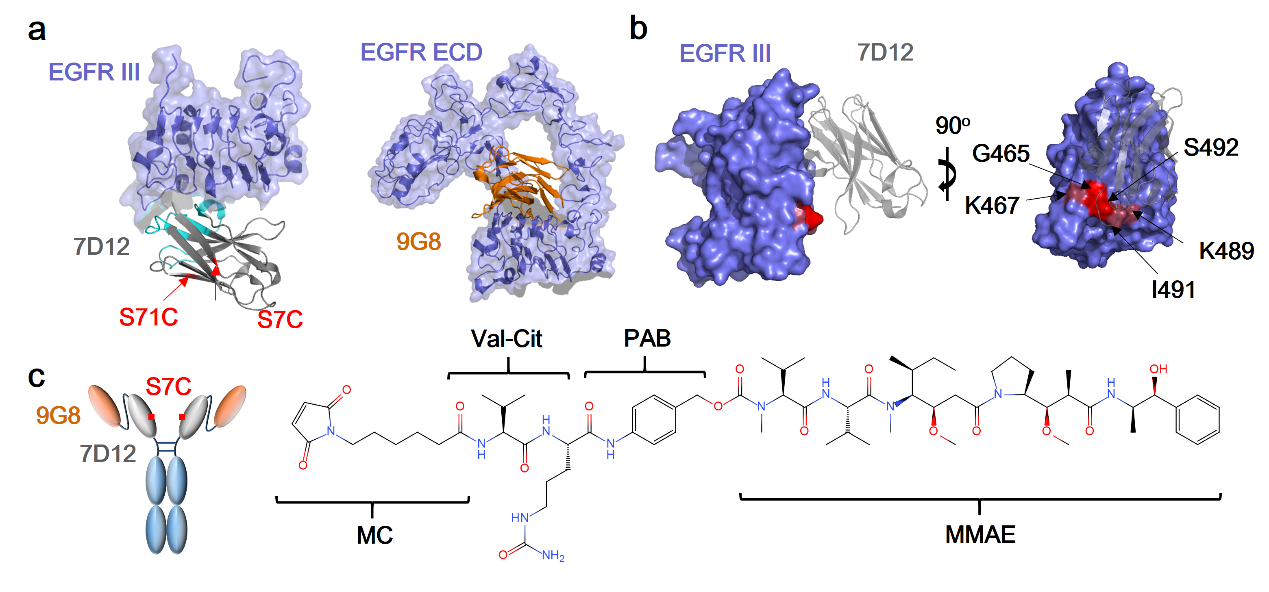


**Supplementary Figure S1. Schematic diagram of nanobodies and EGFR**

(a) Interactions of nanobody 7D12 (gray) and nanobody 9G8 (orange) with EGFR ectodomain (ECD) (blue). Complementarity determining regions (CDRs) of 7D12 are highlighted in light blue, and two introduced cysteine residues S7C and S71C are colored red. Structures of nanobodies and EGFR ECD are adapted from Protein Data Bank (PDB) entries 4KRL, 4KRP by Pymol (Schrödinger).

(b) The binding epitope of nanobody 7D12 and the location of common cetuximab-resistant mutations on EGFR ectodomain III. Two most common cetuximab resistant point mutations S492 and G465 are highlighted in red, and the other point mutations are highlighted dark red.

(c) Schematic diagram of 97m and small molecule cytotoxin Mal-val-cit-PAB-MMAE.


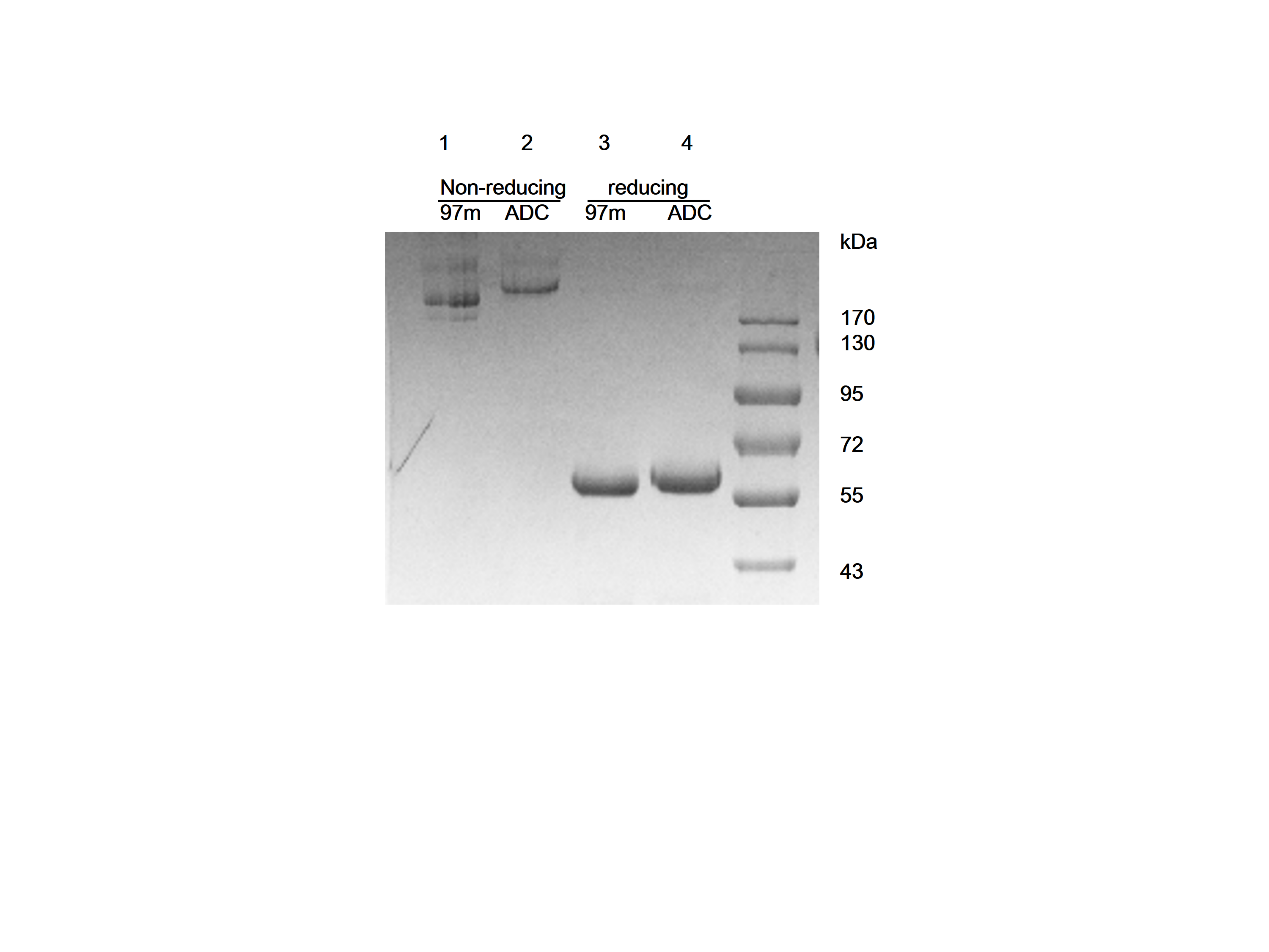


**Supplementary Figure S2. SDS-PAGE analysis of 97m and S7 ADC under non-reducing or reducing conditions.** Lane 1 and 3 represent naked antibody in non-reducing and reducing conditions. Lane 2 and 4 represent ADC in non-reducing and reducing conditions.


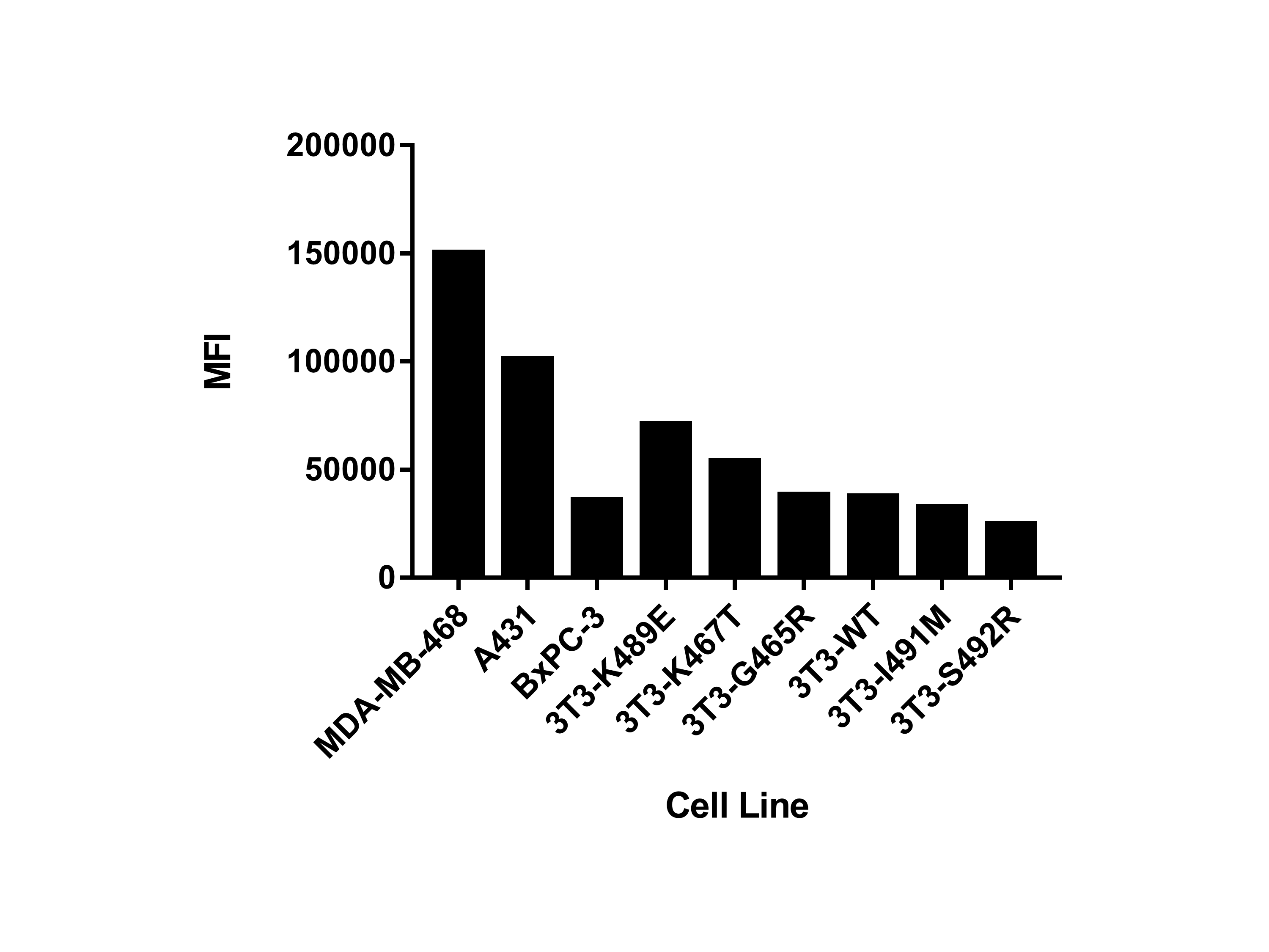


**Supplementary Figure S3. Binding ability of 97m with EGFR or EGFR variants on various cell lines.** Cells were incubated with 97m on ice for 30min and stained with goat anti-Human IgG (H+L) FITC-labeled as secondary antibody.


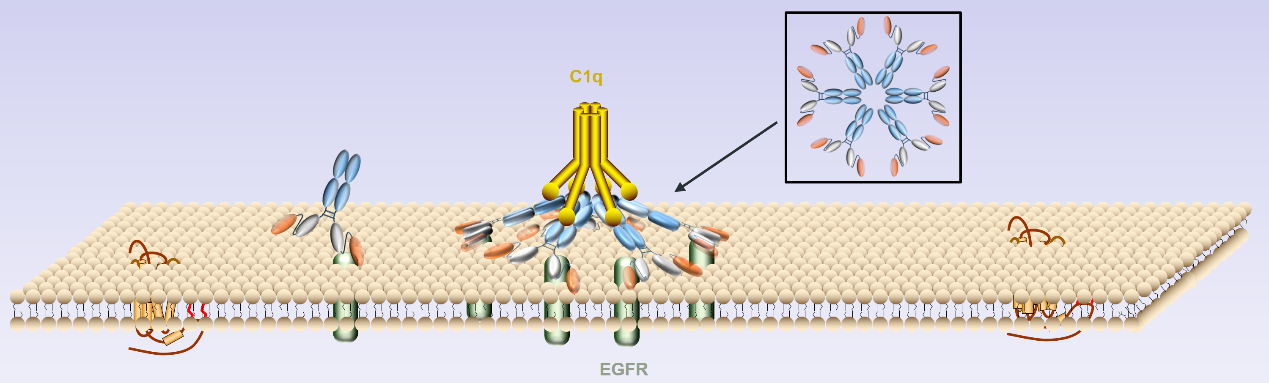


**Supplementary Figure S4. Schematic representation of CDC enhanced hexameric ADC in complex with EGFR and C1q.** The hexamerization-enhancing mutation E430G drives the formation of hexameric biparatopic nanobody (orange, silver and blue) upon its binding with EGFR (green), which promotes hexameric C1q complex (yellow) formation and further potentiates CDC.

**
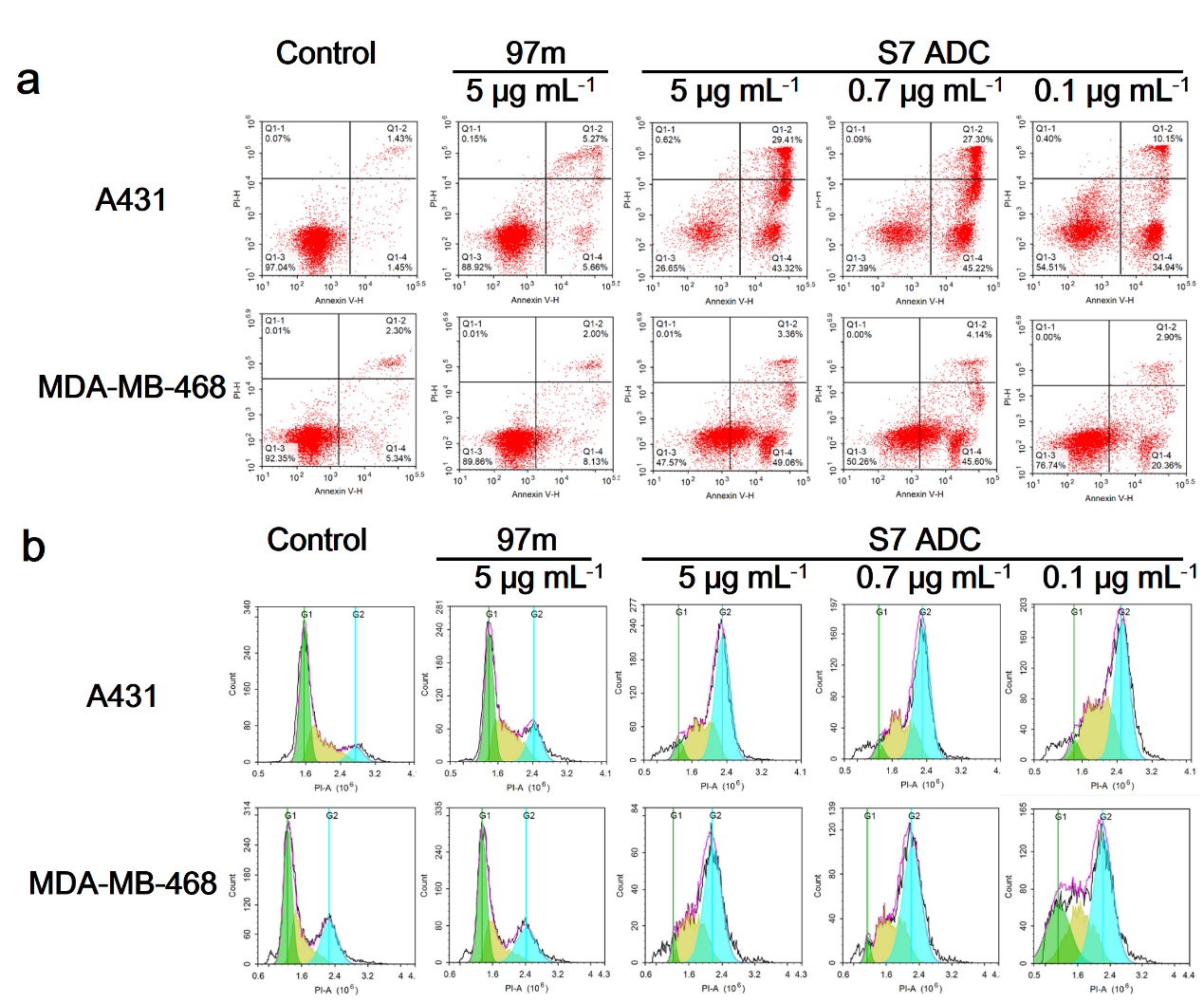
**

**Supplementary Figure S5. Apoptosis-inducing activities and cell cycle blockage by 97m and S7 ADC.**

(a) Apoptosis-inducing activities of 97m and S7 ADC on A431 and MDA-MB-468 tumor cells. The percentages of early apoptotic cells (Annexin V+/PI-) and late apoptotic cells (Annexin V+/PI+) were analyzed by flow cytometry.

(b) Blocking cell cycle of A431 and MDA-MB-468 by 97m and S7 ADC.

G1 phase (green), S phase (yellow) and G2/M phase (blue).


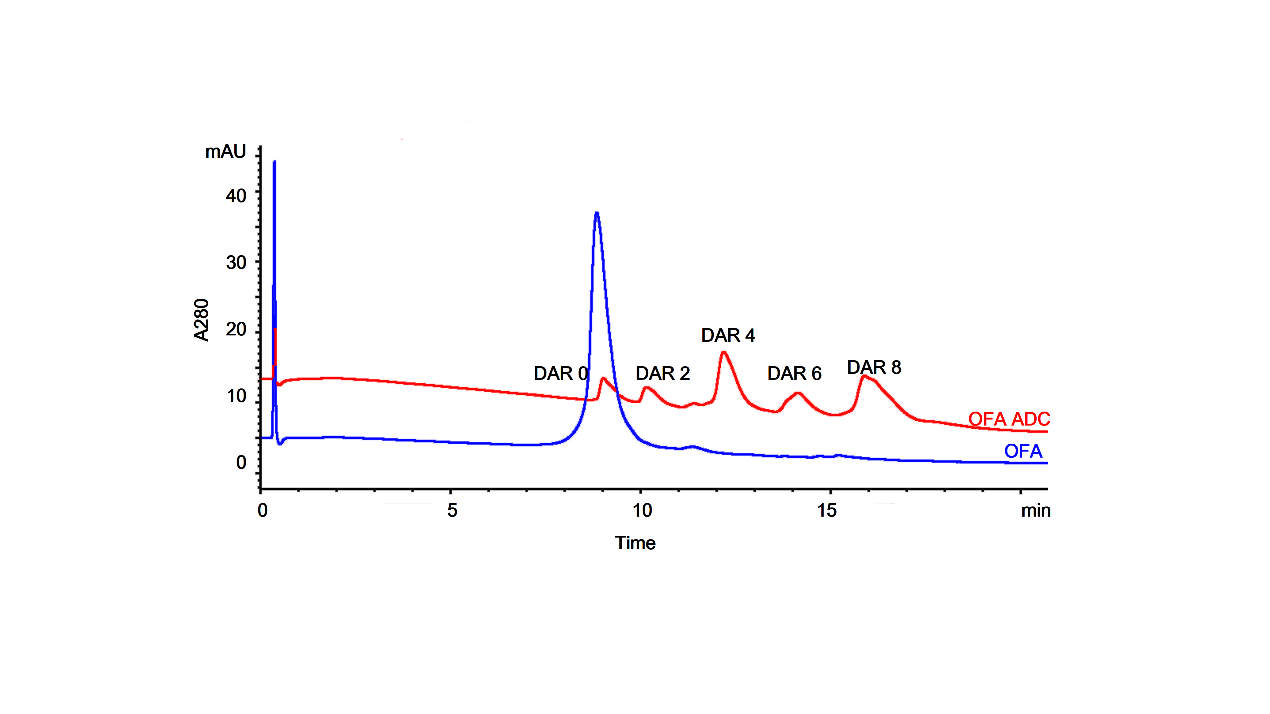
**Supplementary Figure S6. HIC profile of OFA-ADC and ofatumumab.**


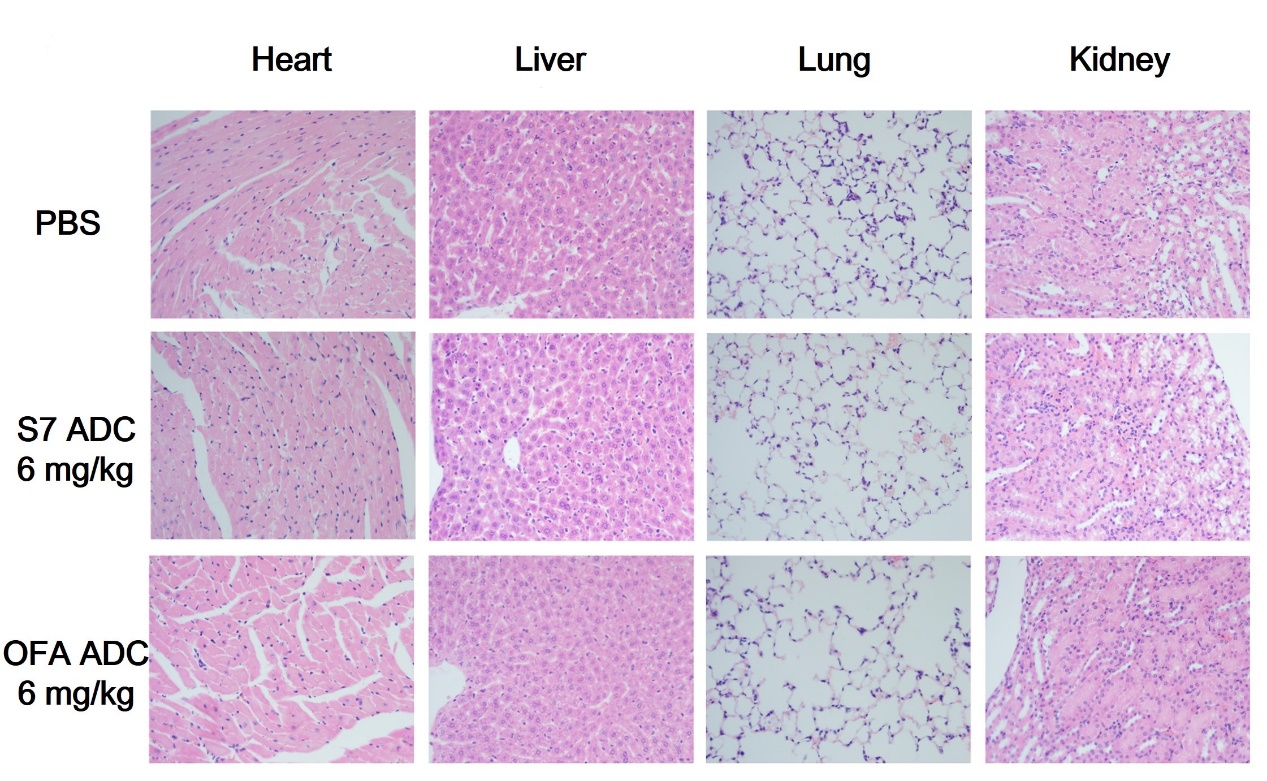


**Supplementary Figure S7. The evaluation of potential acute toxicity via histological examination of primary organs (heart, liver, and kidney).** Magnification: 400×.


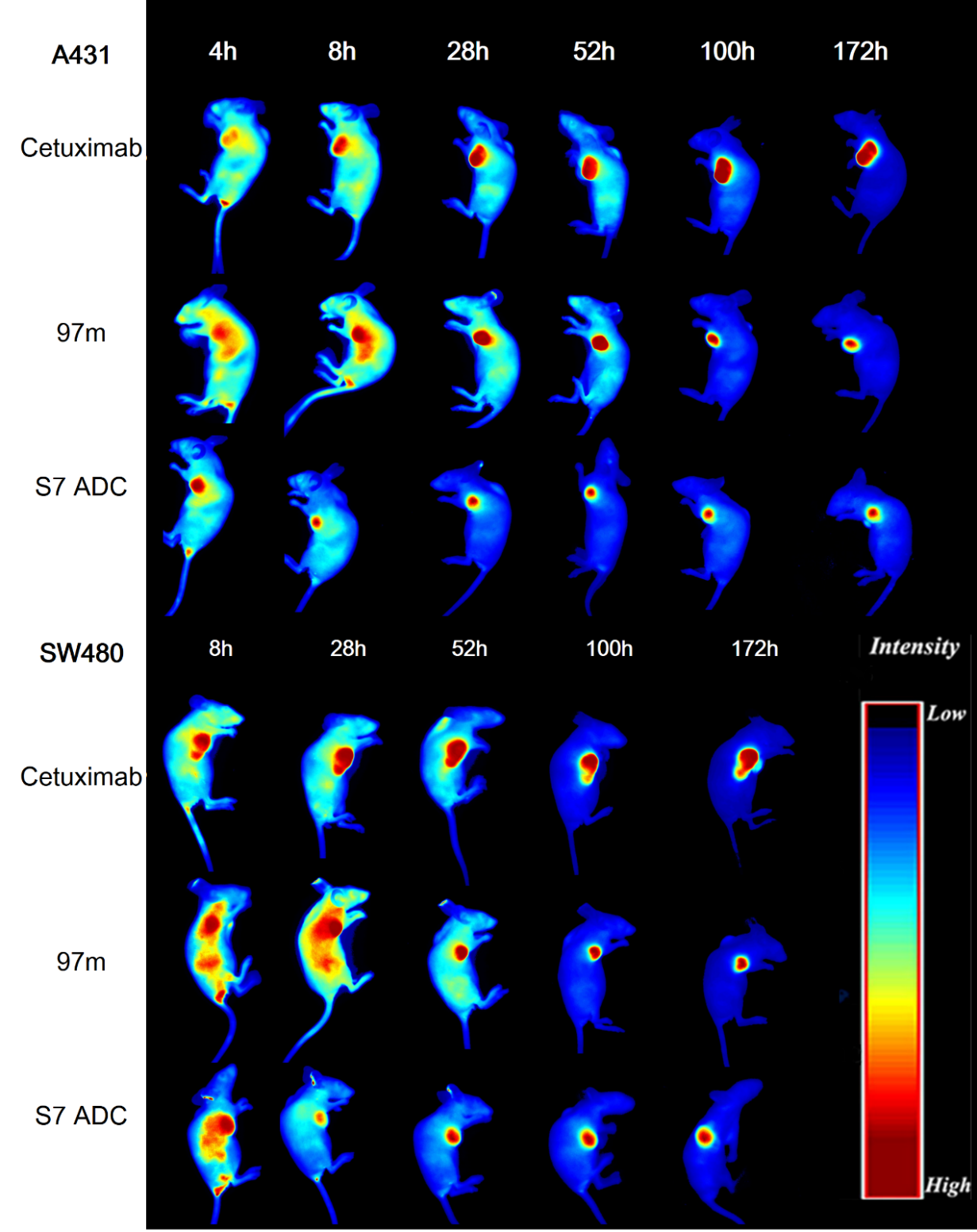


**Supplementary Figure S8. In vivo distribution of the Cy5-labeled cetuximab, 97m and S7 ADC by Maestro in vivo imaging system**.


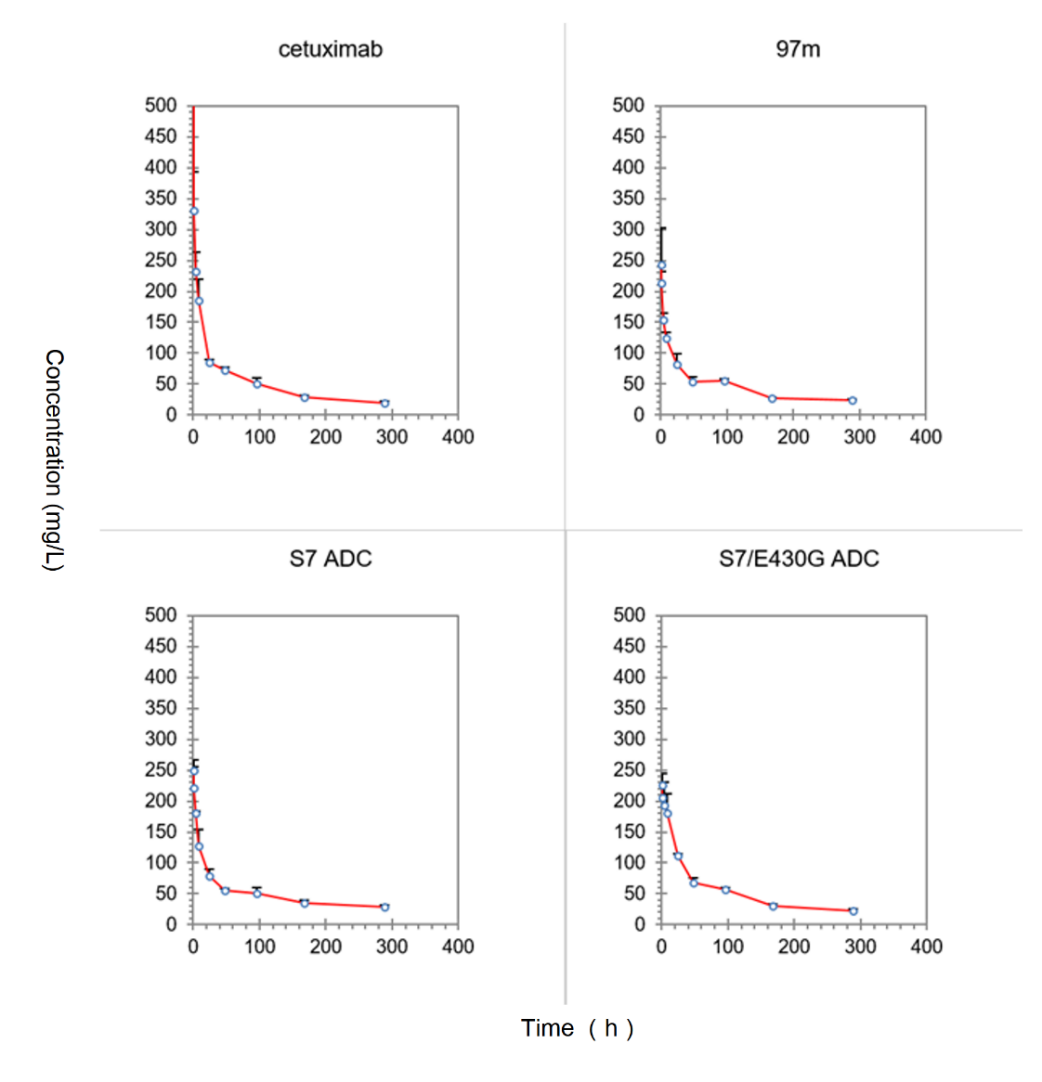


**Supplementary Figure S9. Plasma pharmacokinetics of antibodies and ADCs in mice after single iv injection.**

**Supplementary Table S1. Pharmacokinetic parameters of antibodies and ADCs in Balb/c mice at 5 mg/kg i.v. (n=4). Data are represented as mean ± SD.**

| PK parameters | cetuximab | 97m | S7 ADC | S7/E430G ADC |
| --- | --- | --- | --- | --- |
| AUC_(0-∞)_ (mg/L*h) | 15856.8 ± 415.60 | 14120.59 ± 587.58 | 14346.62 ±1628.82 | 15638.98±566.96 |
| MRT_(0-∞)_ (h) | 111.49 ± 16.00 | 130.23 ± 15.79 | 142.65 ± 21.37 | 107.18 ±6.98 |
| T1/2 (h) | 90.75 ± 10.49 | 98.12 ±17.14 | 109.34 ± 18.43 | 79.65 ± 7.79 |
| Vz (L/kg) | 0.041± 0.004 | 0.05± 0.004 | 0.055 ± 0.007 | 0.037 ± 0.003 |
